# Author Correction: An observational and Mendelian randomisation study on iron status and sepsis

**DOI:** 10.1038/s41598-024-56374-x

**Published:** 2024-03-12

**Authors:** Fergus Hamilton, Ruth Mitchell, Haroon Ahmed, Peter Ghazal, Nicholas J. Timpson

**Affiliations:** 1 MRC Integrative Epidemiology Unit, University of Bristol, Oakfield House, Oakfield Grove, Bristol, BS8 2BN UK; 2 Infection Sciences, North Bristol NHS Trust, Bristol, UK; 3 Division of Population Medicine, Cardiff University Medical School, Cardiff, UK; 4 System Immunity Research Institute, Division of Infection and Immunity, Cardiff University, Cardiff, UK

Correction to: *Scientific Reports * 10.1038/s41598-023-29641-6, published online 17 February 2023

The Funding section in the original version of this Article was incomplete.

“FH’s time was funded by the GW4-CAT Wellcome Doctoral Fellowship Scheme.”

now reads:

“FH’s time was funded by the GW4-CAT Wellcome Doctoral Fellowship Scheme (202802/Z/16/Z).”

The original Article has been corrected.

