# Supplementary material for: An observational and Mendelian randomisation study on iron status and sepsis
Source: Sci Rep. 2023 Feb 17;13:2867. doi: 10.1038/s41598-023-29641-6 (PMC9938246; doi:10.1038/s41598-023-29641-6)

**Supplementary Figure S1:** Flow of participants through the observational study

**
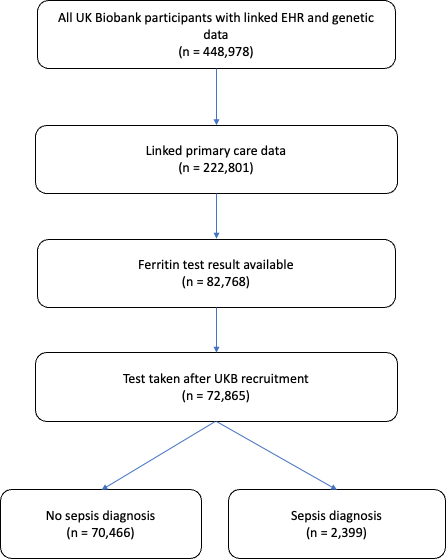
**

**Supplementary Figure S2:** Histogram of Ferritin (log-normalised) for men and women (all tests)


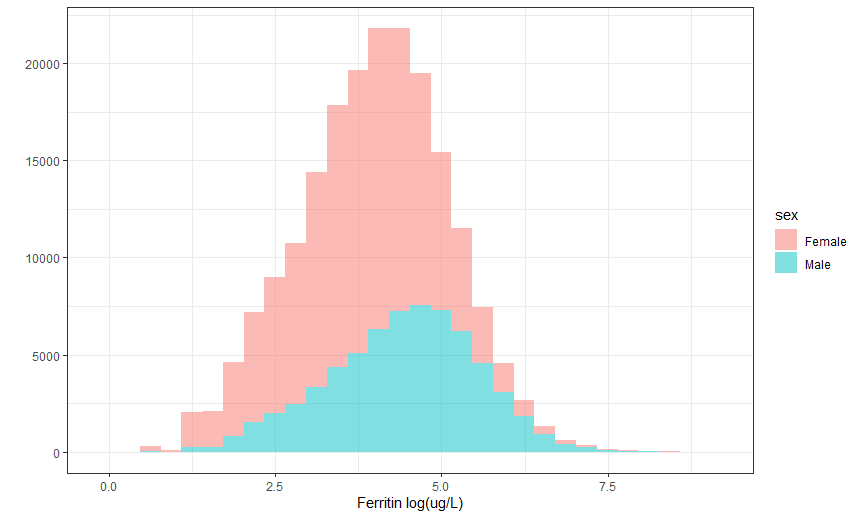


**Supplementary Figure S3:** Restricted cubic spline models of the association between ferritin and sepsis in both men and women. A: Men, B, Women


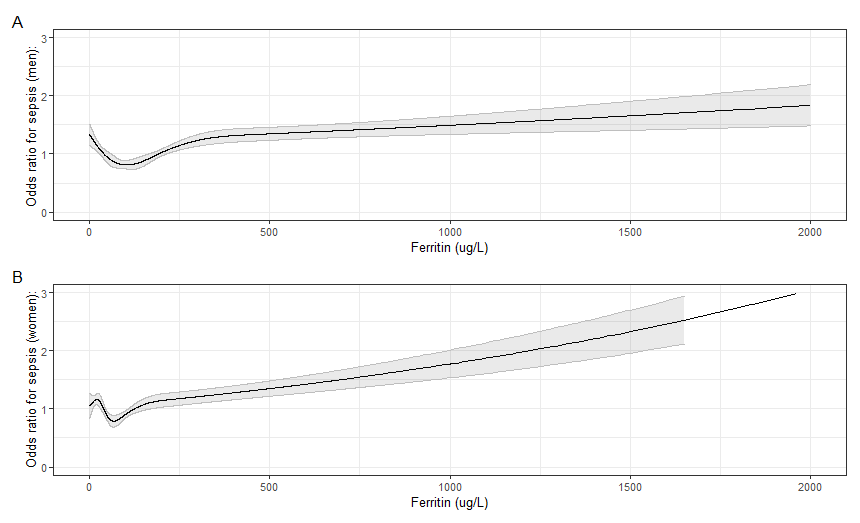


**Supplementary Figure S4:** Quantile-Quantile plot for the under 75 sepsis GWAS


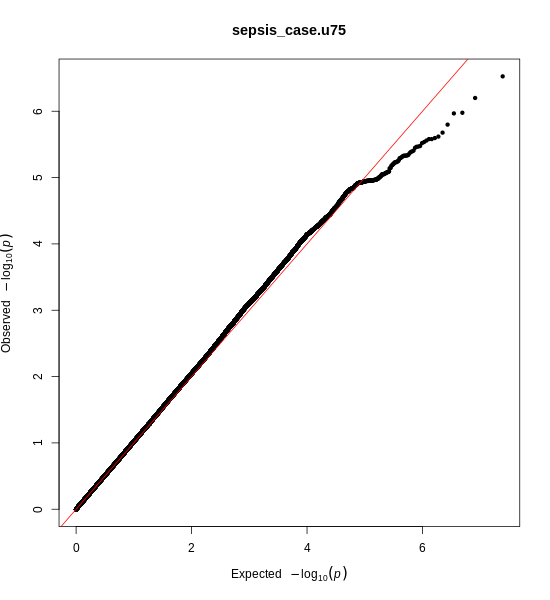


**Supplementary Figure S5:** **Manhattan** **plot for the Sepsis GWAS.**


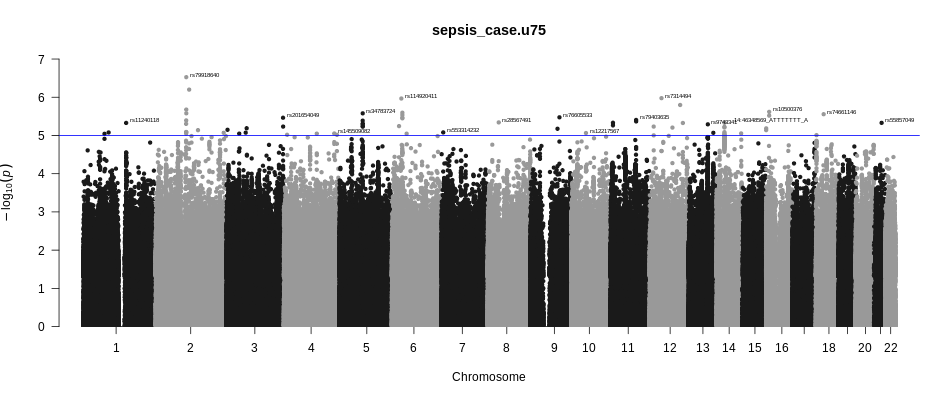


**Supplementary Figure S6:** Scatter plots for all four biomarkers on sepsis (A: TSAT, B: Iron, C: Ferritin, D: TIBC)


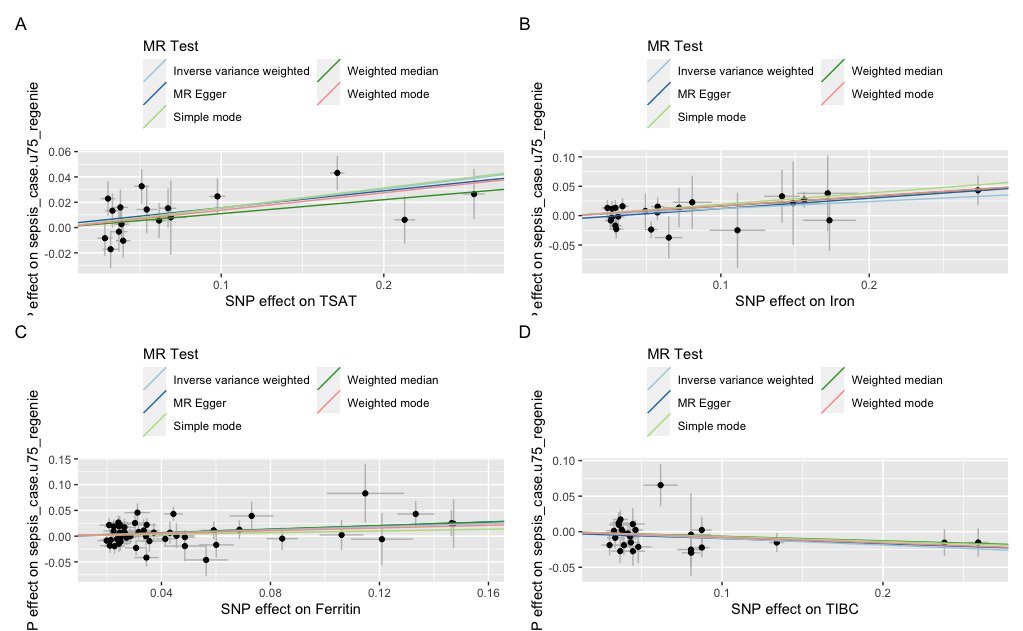


**Supplementary Figure S7:** Scatter plots for FinnGen data (A: TSAT, B: Iron, C: Ferritin, D: TIBC)


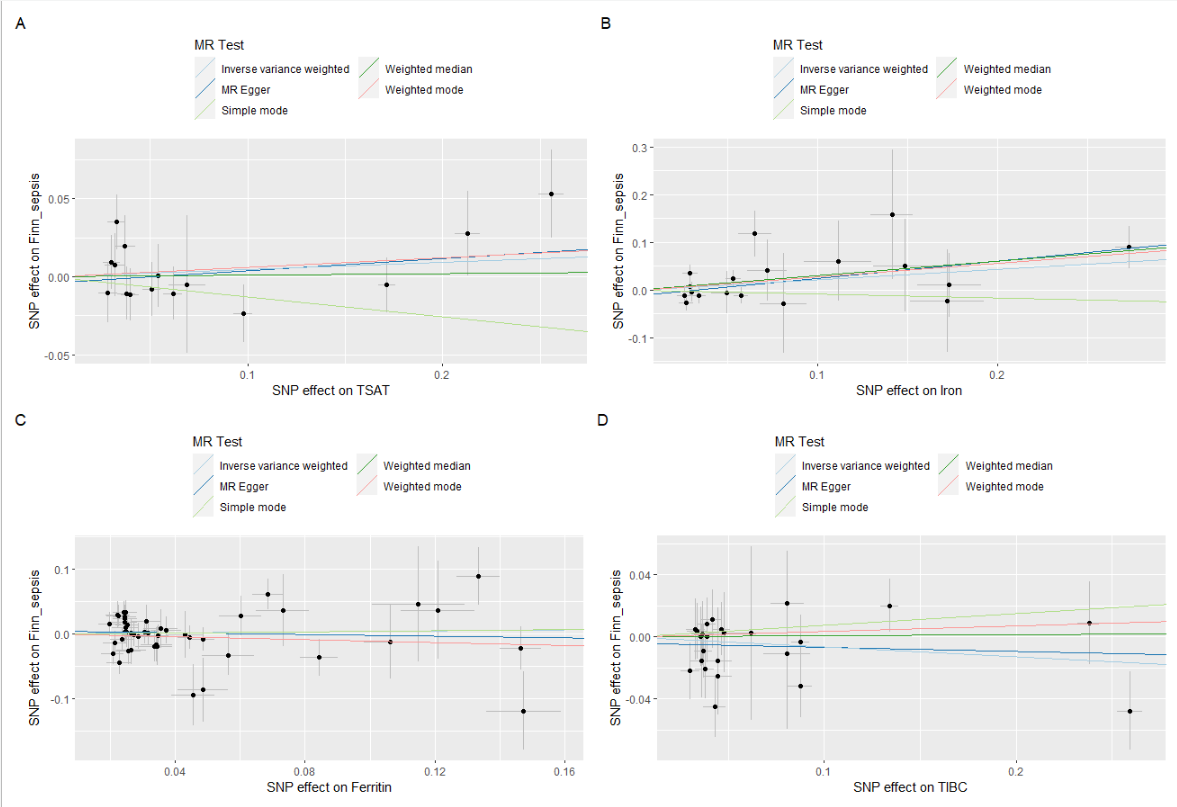


**Supplementary Figure S8:** MR Leave one out analysis. Each row represents the estimates from an IVW analysis removing each individual SNP in turn from the analysis. (A: TSAT, B: Iron, C: Ferritin, D: TIBC)


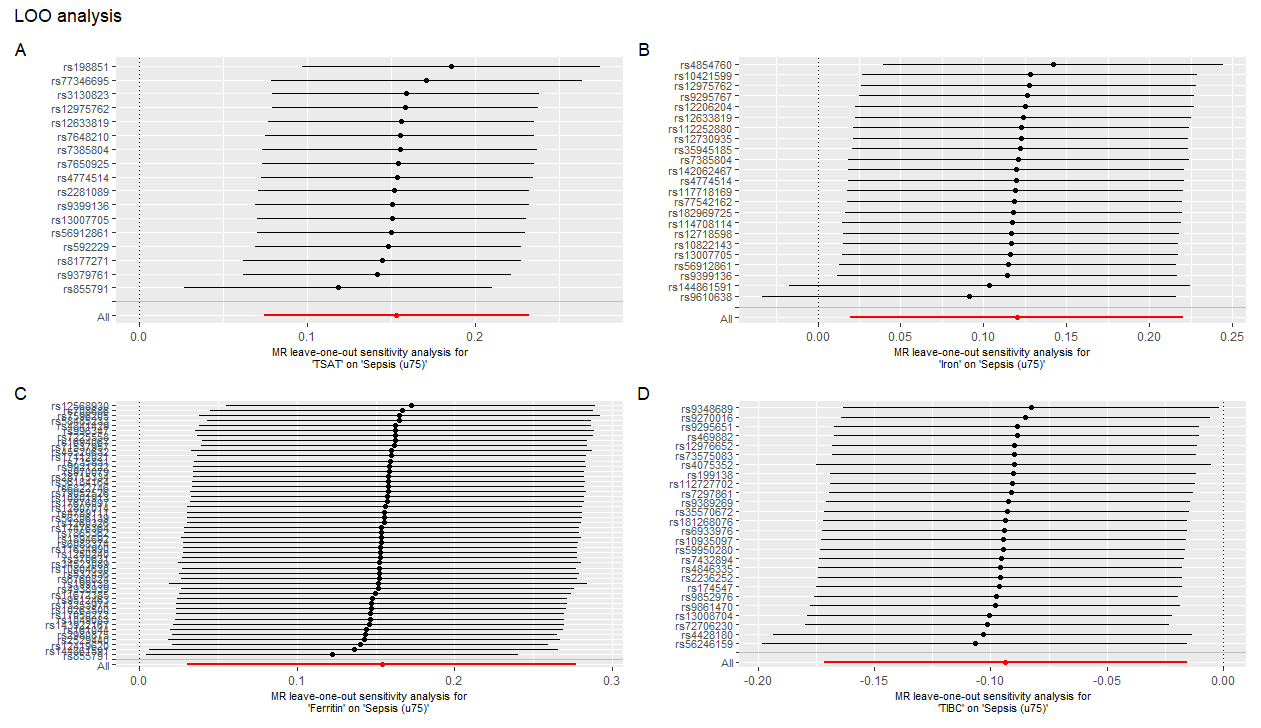


**Supplementary Figure S9: Scatter plots for the TSAT, serum iron, serum ferritin, and TIBC on all cases of sepsis (sensitivity analysis including cases >75 years, A: TSAT, B: Iron, C: Ferritin, D: TIBC)**


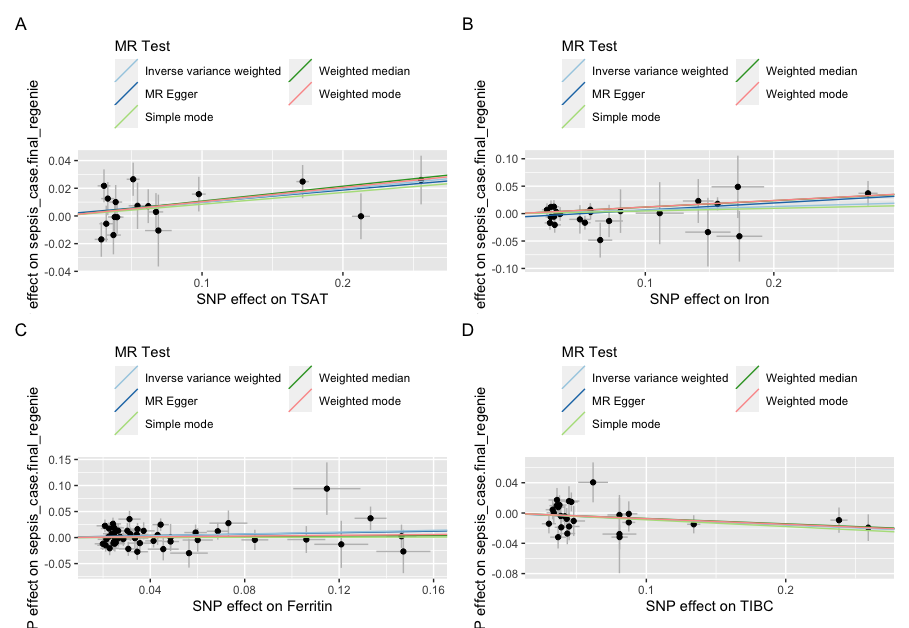

Supplement: Supplementary file 1 — Supplementary Figures. [file 41598_2023_29641_MOESM1_ESM.docx]
