# Supplementary material for: An observational and Mendelian randomisation study on iron status and sepsis
Source: Sci Rep. 2023 Feb 17;13:2867. doi: 10.1038/s41598-023-29641-6 (PMC9938246; doi:10.1038/s41598-023-29641-6)
Supplement: Supplementary file 2 — Supplementary Tables. [file 41598_2023_29641_MOESM2_ESM.docx]

**Supplementary Table S1:** Regression outputs for ferritin. Biobank recruitment centre removed for brevity.

| term | estimate | lower | upper | p.value |
| --- | --- | --- | --- | --- |
| (Intercept) | 0.000 | −1.524 | 1.525 | 0.000 |
| value_100 | 1.050 | 1.036 | 1.064 | 0.000 |
| age_at_entry | 1.065 | 1.059 | 1.071 | 0.000 |
| sexMale | 1.773 | 1.685 | 1.861 | 0.000 |
| bmi | 1.054 | 1.046 | 1.061 | 0.000 |
| liver_disease | 1.503 | 1.274 | 1.731 | 0.000 |
| cancer | 1.892 | 1.783 | 2.001 | 0.000 |
| townsend | 1.036 | 1.022 | 1.050 | 0.000 |
| alcohol_intake_freq.L | 0.876 | 0.454 | 1.297 | 0.537 |
| alcohol_intake_freq.Q | 2.024 | 1.618 | 2.431 | 0.001 |
| alcohol_intake_freq.C | 0.772 | 0.459 | 1.085 | 0.105 |
| alcohol_intake_freq^4 | 1.139 | 0.939 | 1.339 | 0.201 |
| alcohol_intake_freq^5 | 0.902 | 0.766 | 1.038 | 0.136 |
| alcohol_intake_freq^6 | 0.930 | 0.823 | 1.038 | 0.188 |
| as.factor(smoking).L | 0.895 | 0.593 | 1.197 | 0.471 |
| as.factor(smoking).Q | 1.618 | 1.388 | 1.848 | 0.000 |
| as.factor(smoking).C | 0.831 | 0.712 | 0.949 | 0.002 |

**Supplementary Table S2:** Associations between allelic risk score and potential confounders in UK Biobank

| Outcome | beta | Lower 95% CI | Upper 95% CI | P value | n |
| --- | --- | --- | --- | --- | --- |
| Alcohol intake frequency. |  |  |  |  |  |
| Ferritin | 1.008 | 1.003 | 1.013 | 0.003 | 462161 |
| Iron | 1.004 | 0.999 | 1.009 | 0.114 | 462161 |
| TIBC | 1.009 | 1.003 | 1.014 | 0.001 | 462161 |
| TSAT | 1.003 | 0.997 | 1.008 | 0.328 | 462161 |
| Date K76 first reported (other diseases of liver) |  |  |  |  |  |
| Ferritin | 1.019 | 1.001 | 1.038 | 0.037 | 450519/12304(462823) |
| Iron | 1.027 | 1.009 | 1.046 | 0.003 | 450519/12304(462823) |
| TIBC | 0.982 | 0.965 | 1.000 | 0.046 | 450519/12304(462823) |
| TSAT | 1.029 | 1.010 | 1.047 | 0.002 | 450519/12304(462823) |
| Diabetes diagnosed by doctor |  |  |  |  |  |
| Ferritin | 1.013 | 1.000 | 1.027 | 0.057 | 439061/22331(461392) |
| Iron | 1.006 | 0.992 | 1.019 | 0.414 | 439061/22331(461392) |
| TIBC | 1.002 | 0.989 | 1.016 | 0.768 | 439061/22331(461392) |
| TSAT | 1.008 | 0.994 | 1.021 | 0.269 | 439061/22331(461392) |
| Body mass index (BMI) |  |  |  |  |  |
| Ferritin | 0.997 | 0.994 | 1.000 | 0.063 | 461274 |
| Iron | 0.996 | 0.993 | 0.998 | 0.002 | 461274 |
| TIBC | 1.004 | 1.001 | 1.007 | 0.003 | 461274 |
| TSAT | 0.999 | 0.996 | 1.002 | 0.450 | 461274 |
| Date N18 first reported (chronic renal failure) |  |  |  |  |  |
| Ferritin | 0.993 | 0.980 | 1.006 | 0.283 | 439557/23266(462823) |
| Iron | 0.993 | 0.979 | 1.006 | 0.272 | 439557/23266(462823) |
| TIBC | 1.006 | 0.993 | 1.019 | 0.396 | 439557/23266(462823) |
| TSAT | 0.999 | 0.986 | 1.013 | 0.920 | 439557/23266(462823) |
| Cancer diagnosed by doctor |  |  |  |  |  |
| Ferritin | 1.004 | 0.993 | 1.015 | 0.494 | 424971/36155(461126) |
| Iron | 1.011 | 1.000 | 1.022 | 0.046 | 424971/36155(461126) |
| TIBC | 0.994 | 0.983 | 1.005 | 0.278 | 424971/36155(461126) |
| TSAT | 1.014 | 1.003 | 1.025 | 0.010 | 424971/36155(461126) |
| Age at death |  |  |  |  |  |
| Ferritin | 0.998 | 0.987 | 1.009 | 0.669 | 32497 |
| Iron | 1.004 | 0.993 | 1.015 | 0.447 | 32497 |
| TIBC | 1.003 | 0.993 | 1.014 | 0.526 | 32497 |
| Townsend deprivation index at recruitment |  |  |  |  |  |
| Ferritin | 1.000 | 0.998 | 1.003 | 0.774 | 462279 |
| Iron | 0.998 | 0.995 | 1.001 | 0.160 | 462279 |
| TIBC | 0.999 | 0.997 | 1.002 | 0.686 | 462279 |
| TSAT | 0.999 | 0.996 | 1.002 | 0.556 | 462279 |
| Pack years of smoking |  |  |  |  |  |
| Ferritin | 1.001 | 0.995 | 1.006 | 0.803 | 143129 |
| Iron | 1.001 | 0.996 | 1.007 | 0.621 | 143129 |
| TIBC | 0.998 | 0.993 | 1.003 | 0.387 | 143129 |
| TSAT | 1.003 | 0.998 | 1.008 | 0.277 | 143129 |

**Supplementary Table 3:** MR estimates from alternative meta-analytic strategies (MR Egger, Weighted Median, IVW, and MR-PRESSO) for each biomarker.

| **Meta-analytic approach** | **Beta** | **Standard error** | **P value** |
| --- | --- | --- | --- |
| *TSAT* | | | |
| MR Egger | 0.13368717 | 0.06318653 | 0.0515038915 |
| Weighted median | 0.11034571 | 0.05625426 | 0.0498145422 |
| Inverse variance weighted | 0.15282230 | 0.04019315 | 0.0001434181 |
| MR-PRESSO | 0.15282230 | 0.03979998 | 0.0014462219 |
| *Iron* | | | |
| MR Egger | 0.17608145 | 0.07503957 | 0.0288394404 |
| Weighted median | 0.16345201 | 0.06278099 | 0.0092269937 |
| Inverse variance weighted | 0.12001259 | 0.05139021 | 0.0195267032 |
| MR-PRESSO | 0.12001259 | 0.04358092 | 0.0115869427 |
| *Ferritin* | | | |
| MR Egger | 0.18211621 | 0.12071923 | 0.1379573205 |
| Weighted median | 0.17105931 | 0.09187505 | 0.0626215592 |
| Inverse variance weighted | 0.15373408 | 0.06283945 | 0.0144267646 |
| MR-PRESSO | 0.15373408 | 0.06283945 | 0.0180619159 |
| *TIBC* | | | |
| MR Egger | -0.07393453 | 0.06198034 | 0.2445801174 |
| Weighted median | -0.06425524 | 0.05258048 | 0.2216940210 |
| Inverse variance weighted | -0.09366362 | 0.03983405 | 0.0187056505 |
| MR-PRESSO | -0.09366362 | 0.03676677 | 0.0173789952 |

**Supplementary Table S4**: All case analysis using UK Biobank. This analysis reports the outcomes using sepsis diagnosed at all ages rather than <75 (our primary analysis)

|  | Odds ratio | Lower 95% CI | Upper 95% CI | P value |
| --- | --- | --- | --- | --- |
| *UK Biobank (all cases)* | | | | |
| Iron | 1.07 | 0.98 | 1.16 | 0.16 |
| TIBC | 0.92 | 0.86 | 0.99 | 0.03 |
| TSAT | 1.10 | 1.03 | 1.17 | 0.01 |
| Ferritin | 1.09 | 0.99 | 1.20 | 0.11 |
| *Combined – IVW meta-analysis* | | | | |
| Iron | 1.12 | 1.01 | 1.19 | 0.04 |
| TIBC | 0.93 | 0.88 | 0.98 | 0.01 |
| TSAT | 1.09 | 1.02 | 1.15 | 0.01 |
| Ferritin | 1.08 | 0.98 | 1.18 | 0.11 |
